# Type 2 Diabetes-Related Health Economic Impact Associated with Increased Whole Grains Consumption among Adults in Finland

**DOI:** 10.3390/nu13103583

**Published:** 2021-10-13

**Authors:** Janne Martikainen, Kari Jalkanen, Jari Heiskanen, Piia Lavikainen, Markku Peltonen, Tiina Laatikainen, Jaana Lindström

**Affiliations:** 1 School of Pharmacy, University of Eastern Finland, 70211 Kuopio, Finland; kari.jalkanen@uef.fi (K.J.); jari.heiskanen@uef.fi (J.H.); piia.lavikainen@uef.fi (P.L.); 2 Department of Public Health and Welfare, Finnish Institute for Health and Welfare, 00271 Helsinki, Finland; markku.peltonen@thl.fi (M.P.); tiina.laatikainen@thl.fi (T.L.); jaana.lindstrom@thl.fi (J.L.); 3 Institute of Public Health and Clinical Nutrition, University of Eastern Finland, 70211 Kuopio, Finland; 4 Joint Municipal Authority for North Karelia Health and Social Services (Siun Sote), 80210 Joensuu, Finland

**Keywords:** whole grains, diabetes, healthcare costs, cost saving analysis, quality-adjusted life years, nutrition economics

## Abstract

The prevalence of type 2 diabetes (T2D) is increasing rapidly worldwide. A healthy diet supporting the control of energy intake and body weight has major importance in the prevention of T2D. For example, a high intake of whole grain foods (WGF) has been shown to be inversely associated with risk for T2D. The objective of the study was to estimate the expected health economic impacts of increased WGF consumption to decrease the incidence of T2D in the Finnish adult population. A health economic model utilizing data from multiple national databases and published scientific literature was constructed to estimate these population-level health economic consequences. Among the adult Finnish population, increased WGF consumption could reduce T2D-related costs between 286€ and 989€ million during the next 10-year time horizon depending on the applied scenario (i.e., a 10%-unit increase in a proportion of daily WGF users, an increased number (i.e., two or more) of WGF servings a day, or alternatively a combination of these scenarios). Over the next 20–30 years, a population-wide increase in WGF consumption could lead to much higher benefits. Furthermore, depending on the applied scenario, between 1323 and 154,094 quality-adjusted life years (QALYs) could be gained at the population level due to decreased T2D-related morbidity and mortality during the next 10 to 30 years. The results indicate that even when the current level of daily WGF consumption is already at a relatively high-level in a global context, increased WGF consumption could lead to important health gains and savings in the Finnish adult population.

## 1. Introduction

Type 2 diabetes (T2D) is one of the most common metabolic diseases and represents a leading cause of morbidity and mortality because of its related micro- and macrovascular complications. The number of people with T2D is expected to increase dramatically in the next decades [1]. Overweight and obesity associated with excess energy intake, Western dietary habits, and low physical activity are the major determinants of the rise in T2D prevalence [2,3]. As a result of this adverse development, global and regional diabetes-related health expenditures are expected to grow significantly [1]. 

Observational evidence has suggested that WGFs are beneficial in regard to T2D risk [4,5,6,7,8,9,10], and the finding has also been supported by an intervention study that has emphasized the consumption of WGFs as a way to increase dietary fiber intake [11,12].

In Finland, daily WGF consumption is relatively high compared with many other countries [13]. Currently around 76% and 67% of Finnish men and women, respectively, reach the daily goal of dietary fiber intake as recommended by the national nutritional guidelines [14]. In addition, fiber-rich WGFs contain other components, which may offer important beneficial effects including balanced glucose metabolism [15,16,17] and many other health conditions [18,19]. Thus, the formulation and promotion of WGFs may have significant health and economic consequences regarding the prevention of T2D at the population level, as indicated by previous modeling studies from Australia and Canada [20,21,22]. To highlight the potential of such policies in the Finnish setting, the aim of the present study was to evaluate the savings potential as well as health impacts in terms of quality-adjusted life years (QALYs) of increasing daily WGF consumption as a method to decrease the incidence of T2D and its consequences in the Finnish adult population.

## 2. Materials and Methods

### 2.1. Model Overview

**Figure 1:**
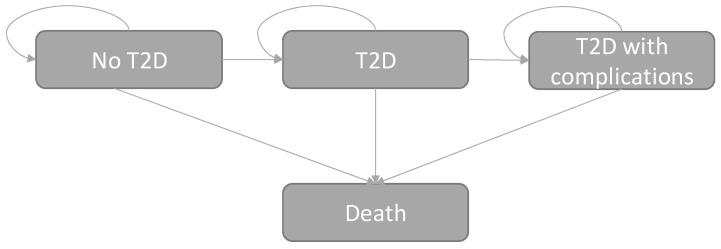
Schematic presentation of the applied Markov model showing the considered health states for the prevention of T2D. Arrows indicate possible transitions between health states in the model.

To estimate the expected health and economic consequences of increased daily WGFs consumption among the Finnish adult population, a health economic model utilizing data from multiple national databases and published scientific literature was constructed. The developed Markov-type cohort model included four mutually exclusive health states (i.e., No T2D, T2D, T2D with complications, and death) to project the expected incidence of T2D and its complications based on the observed population risk factor levels of T2D in the national FinHealth 2017 study [23]. The year 2017 was applied as a baseline year in the present study. The developed model is schematically depicted in Figure 1. The graphical scheme of the study design is provided in Supplementary Figure S1.

The model was populated with the characteristics of the Finnish adults aged 30–79 years without T2D at baseline (*n* = 2.97 million Finnish adults in 2017) as well as with the age- and sex-specific risk of T2D development during the next 10 years measured as the Finnish Diabetes Risk Score (FINDRISC) [24]. The FINDRISC is a validated questionnaire used to estimate the 10-year risk of developing T2D based on sex, age, body mass index (kg/m^2^), use of blood pressure medication, history of high blood glucose, physical activity, daily consumption of vegetables, fruits, or berries, as well as family history of diabetes. In the present study, the FINDRISC score was divided in five age- and sex-specific categories (i.e., from low risk to very high risk) indicating the 10-year risk of T2D (see Supplementary Table S1 for details). Other baseline characteristics applied as the input parameters of the model are described in Table 1. In the developed model, this hypothetical cohort of Finnish adults without T2D at baseline were at risk of developing T2D or T2D-related complications (if already having T2D), or they might survive to the next year (i.e., 1-year cycle length was applied in the model) without any event. Finally, the developed model was used to estimate the expected number of new T2D cases and associated consequences (in terms of costs and QALYs) with and without expected increase in WGF consumption using 10-year, 20-year, and 30-year time horizons. All analyses were implemented in R using the HEEMOD package, which is an R toolset for health economic modeling [25]. 

#### 2.1.1. Baseline Risk of T2D

In the health economic modeling, parametric survival regression models are commonly used to extrapolate event risks over the actual follow-up time [27]. In the present study, a parametric survival regression model was used to estimate the risk of T2D based on the national FINRISK data (*n* = 9512) linked with 10-year register-based follow-up data [28]. The Weibull survival regression model, which provided the most reliable fit (i.e., based on applied Akaike and Bayesian information criteria and visual inspections) to the available data, was used to estimate the relationship between baseline age, sex, and FINDRISC categories and the incidence of T2D (indicated as new reimbursement rights and/or the first purchases for T2D medicines observed from the national medicine reimbursement registry maintained by the Social Insurance Institution of Finland) over 10-year follow-up. Annual transition probabilities (conditional on age, sex, and FINDRISC categories) applied in the developed Markov model were estimated based on these estimated incidence rates. The coefficients of the Weibull regression for incidence of T2D are shown in Supplementary Table S2.

#### 2.1.2. Risk of T2D with Complications

To estimate the risk of T2D-related complications in persons with newly diagnosed T2D, a real-world dataset based on electronic health record (EHR) data of patients with T2D and living in the county of North Karelia in Finland was applied [29]. For the purposes of the present study, the data of patients with a newly diagnosed T2D between 2011 and 2012 (*n* = 1151) were extracted from the dataset to estimate the development of T2D-related complications after the diagnosis of T2D. The data were available until December 2019 with the longest follow-up duration of 9.0 years. To estimate the risk of T2D-related complications, all T2D-related renal, eye, cardiovascular, cerebrovascular, neuropathic, and foot complications (see Supplementary Table S3 for details), as well as date of diagnoses were extracted from the data, and a Weibull survival regression model was fitted to estimate the annual rates of complications based on sex and baseline age. Annual age- and sex-specific transition probabilities applied in the developed Markov model were estimated based on these estimated complication rates. The coefficients of the Weibull regression for incidence of T2D with complications are shown in Supplementary Table S4.

#### 2.1.3. Risk of Death

The national all-cause life tables for men and women were used to characterize the risk of death conditional on age and sex [30]. In addition, deaths in the modeled “T2D” and “T2D with complications” health states were adjusted to consider the increased risk of death in those health states by applying previously published HRs [31,32]. To avoid the risk of double counting, the increased WGF consumption was assumed to have no direct impact on all-cause mortality.

#### 2.1.4. Estimating the Effects of Increased Whole Grain Intake in the Reduction of T2D

For the purposes of the present study, the developed model was calibrated to correspond with the observed 10-year incidence of T2D in the Finnish adults reporting no daily WGF consumption (i.e., no daily use of rye bread, porridge, or mixed bread) in the FINRISK study. Based on the FINRISK register-enriched follow-up dataset, the average observed 10-year incidence of T2D was 7.69% in this subpopulation. This approach enabled the use of the results of a recent meta-analysis studying the dose–response association between the daily WGF intake (measured as servings a day) and the long-term risk reduction of T2D (using no daily use of WGF as a reference) with a total of 4,618,796 person years of follow-up and with the average follow-up time of 24 years [10]. According to the multivariable-adjusted study results, one serving of WGF was expected to reduce the risk for T2D by 27% (Hazard Ratio (HR) 0.73, 95%CI 0.72–0.74), whereas two or more servings of WGFs were expected to reduce the risk of developing T2D by 35% (HR 0.65, 95%CI 0.61–0.68). Since the applied baseline risk of T2D was defined to represent the risk among those with no regular daily WGF consumption, the transition probabilities were adjusted by applying weighted HR estimates to correspond with a proportion (i.e., 69.5% according to the applied definition in the present study) of Finnish adults using at least one WGF serving a day as observed in the applied FINRISK dataset.

In the present study, three alternative scenarios were studied: (I) 10%-unit increase in the proportion of the Finnish adult population using at least one WGF serving a day, (II) one or more additional WGF servings a day [33] among those who already use at least one WGF serving a day, and (III) a scenario combining scenarios I and II. In addition, to simplify the analysis, the full effect of increasing daily WGF intake was assumed to be achieved immediately and to persist over time.

#### 2.1.5. Cost Data

A limited societal perspective was applied in the present study, since direct non-medical costs, such as travel costs associated with the utilization of health care services, were not considered in the present study due to limited data availability. The estimates of additional health care and T2D-related productivity loss costs (i.e., costs associated with sick leaves, premature retirements, and premature deaths) were obtained from the national cost reports [34,35,36]. These estimates included both the additional secondary health care costs and T2D-related productivity losses estimated using the Finnish national registries and a case-control study design (with adjustments for age, sex, and living area). In the model, T2D-related productivity losses were applied to adults with T2D below the average age of retirement (i.e., 65 years of age). 

In addition, the additional primary care costs due to T2D were estimated using the above-mentioned EHR dataset (*n* = 1151) from the county of North Karelia by applying a case-control study design with adjustments for age, sex, and living area. In addition, the annual average (per-person) T2D medication (ATC-code A10) costs were obtained from the national medicine statistics maintained by the Social Insurance Institution of Finland. Finally, all costs were adjusted to the 2019 price level using the official health care price index determined by Statistics Finland. All unit cost estimates are summarized in Table 2. In the base-case analysis, a 3% discount rate per year was applied for costs and QALYs in accordance with the national HTA guidelines [37].

#### 2.1.6. Utility Weights

The published population-level EQ-5D-3L utility values (stratified by age and sex) were applied to represent the average health-related quality of life in the target population [38,39]. EQ-5D-3L-based disutility weights associated with T2D and its complications were also obtained from previously published studies [40,41,42,43,44]. Disutility associated with T2D with complications was estimated as a weighted average, where disutility values associated with a single complication were weighted by their observed incidences between years 2000 and 2017 in Finland [45]. The applied utility and clinical data are described in Table 3. 

#### 2.1.7. Sensitivity Analyses

To test the robustness of different assumptions related to modeling, different deterministic one-way sensitivity analyses were conducted. The results of these sensitivity analyses were presented in the form of a tornado diagram. In addition, parameter uncertainty associated with the model inputs was studied by using probabilistic sensitivity analysis (PSA) with 1000 random iteration rounds [27,46]. The correlation structure between the Weibull regression coefficients was also taken into consideration, and the regression coefficients were assumed to be normally distributed (Supplementary Table S5). Results of the PSA were presented on the X-Y plane demonstrating the joint distribution of cumulative savings and QALYs gained conditional on the selected time horizon. In addition, the probabilities of cumulative savings (with and without T2D-related productivity losses) given the available data were estimated based on the obtained PSA results [39]. 

**Table 2:** Costs applied in the Markov model, their distributions, and the values used to estimate the distributions. Costs before 2019 have been discounted to the latest values.

Parameter	Value (Variation) *	Distribution	Distribution Values Used in PSA Mean (SE)	Source
Additional health care costs of T2D excluding basic health care	3315 € (±25%)	Gamma	3315€ (423€)	[35]
Cost of T2D complications	4401€ (±25%)	Gamma	4401€ (561€)	[34]
Costs from productivity losses due to T2D	7632€ (±25%)	Gamma	7632€ (974€)	[36]
Additional T2D health care costs for primary health care	Men 562 € (SD 587€) Women 542 € (SD 649 €)	Gamma	Men 562€ (9.53€) Women 542€ (9.82€)	Based on own results
Additional medication costs of T2D	584 € (±25%)	Gamma	584€ (74€)	[47]

* For variables without available confidence interval, a variation of ± 25% has been used as an estimate. PSA; Probabilistic Sensitivity Analysis.

**Table 3:** Parameters applied in the Markov model, their distributions, and the values used to estimate the distributions.

Parameter	Value (Variation) *	Distribution Applied in PSA	Distribution Values Used in PSA Mean (SE)		Source
T2D-specific mortality risk, Hazard ratio (95% CI)	Women HR 2.47 (2.42–3.06) Men HR 1.93 (1.79–2.07)	Lognormal	2.47 (0.04) 1.93 (0.05)		[32]
Mortality risk associated with T2D with complications, Hazard ratio (95% CI)	HR 2.36 (1.70–3.29)	Lognormal	2.36 (0.41)		[31]
All-cause mortality	Based on age and sex	-	-		[30]
Utilities					
Baseline utilities (EQ-5D-3L)		Beta	Alpha	Beta	[39]
			(value)	(value)	
	Women				
	(Age, Utility, SE)				
	30–44 0.906 (0.003)		8573	889	
	45–54 0.865 (0.005)		4040	631	
	55–64 0.810 (0.006)		3463	812	
	65+ 0.770 (0.008)		2130	636	
	Men		Men	Men	
	(Age, Utility, SE)				
	30–44 0.917 (0.003)		7755	702	
	45–54 0.876 (0.005)		3806	539	
	55–64 0.821 (0.006)		3351	731	
	65+ 0.781 (0.008)		2087	585	
Disutility of T2D (EQ-5D-3L) (SE)	0.041	Beta	Alpha	Beta	[38]
	(0.012)		11.19	261.9	
Weighted disutility of T2D complications (EQ-5D-3L)	0.119 (±25%)	Beta	0.119 (0.015)		Disutility values of individual complications [40,41,42,43,44] Proportion of complications [45]

* For variables without available confidence interval, a variation of ±25% has been used as an estimate. PSA; Probabilistic Sensitivity Analysis.

## 3. Results

### 3.1. Population Results

Based on the simulation results of the calibrated model when assuming no change in the current daily use of WGFs, the expected discounted total T2D-related costs among the Finnish adults aged 30–79 (*n* = 297 million) were 8032€, 25,867€, and 46,491€ million during the applied 10-year, 20-year, and 30-year time horizons, respectively. Assumed increased WGF consumption could reduce these total costs between 286€ and 989€ million during the next 10-year time horizon depending on the applied scenario. Over the next 20 to 30 years, a population-wide increase in WGF consumption could potentially lead to much higher cumulative savings in the health care sector and productivity gains in the society, as shown in Table 4. Furthermore, depending on the applied scenario, a total of 1323 to 154,094 QALYs could be gained at the population level due to decreased T2D-related morbidity and mortality at the population level during the next 10 to 30 years (Table 5). 

### 3.2. Results of One-Way Sensitivity Analyses

**Figure 2:**
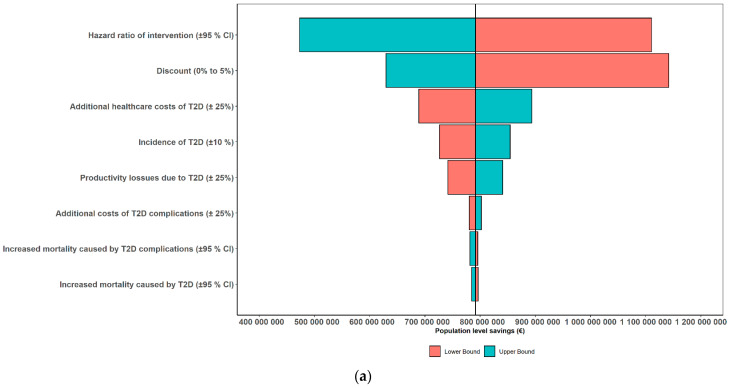

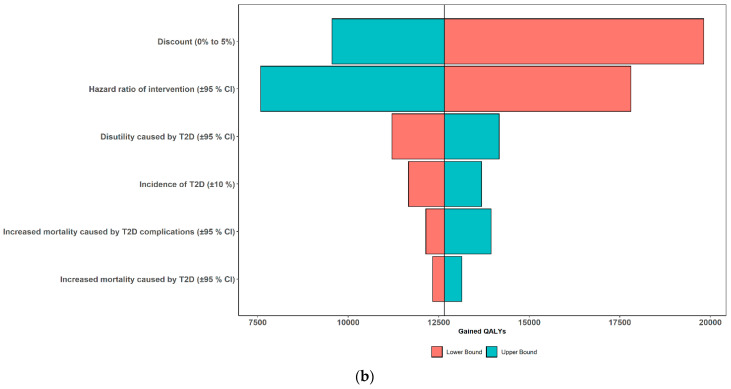
Tornado diagrams showing the results of one-way sensitivity analyses in terms of (**a**) cost savings and (**b**) additional QALYs in Scenario I (i.e., 10%-unit increase in the proportion of Finnish adults with the daily use of whole grain foods) with the 20-year timeframe as an example.

In one-way sensitivity analyses, when using the 20-year time horizon as an example, the largest effect on the results was the effectiveness of intervention and applied discount rate (Figure 2a,b). Savings variated from 473€ to 1110€ million and gained QALYs variated from 7583 to 17,812 when the effectiveness estimate was varied according to its 95%CIs. Changing the discount rate from 0% to 5%, the savings varied from 629€ to 1140€ million, and the gained QALYs variated from 9551 to 19,821. Other studied model parameters had a modest or small effect on the potential population level savings in all the studied scenarios. 

### 3.3. Results of Probabilistic Sensitivity Analysis

**Figure 3:**
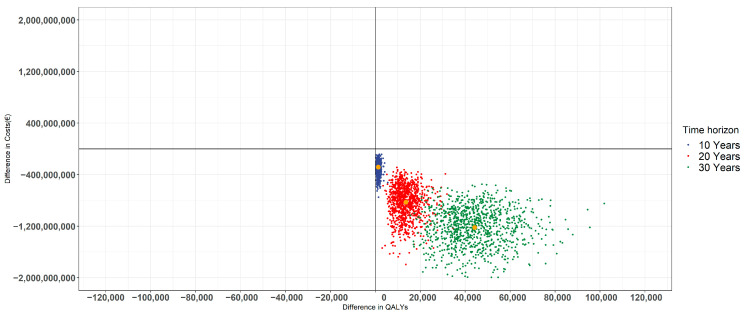
Results of the probabilistic sensitivity analysis showing the impact of applied time horizon on the distribution of expected population-level cost savings and gained QALYs on the X-Y-plane using Scenario I (current situation vs. a 10%-unit increase in the proportion of adult population using at least one whole grain servings a day) as an example. Blue, red, and green colors stand for 10-year, 20-year, and 30-year time horizons, respectively.

**Figure 4:**
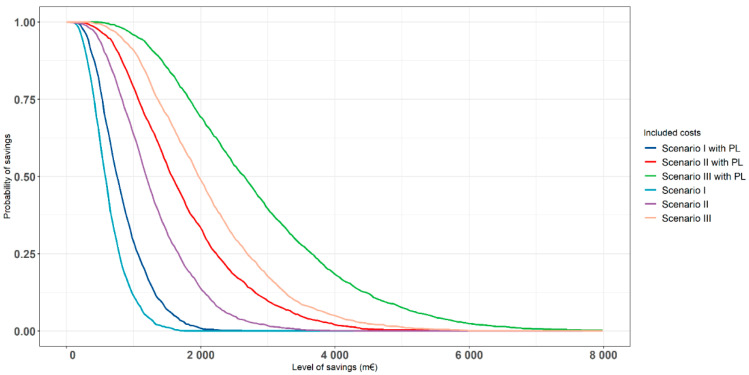
Probability of cumulative savings (with and without T2D-related productivity losses) in the modeled scenarios when applying the 20-year time horizon (2017 as a baseline year). Scenario I: a 10%-unit increase in the Finnish population using at least one whole grain food serving a day, Scenario II: one or more additional whole grain servings a day among those who already use at least one whole grain serving a day, and Scenario III: the combination of Scenarios I and II. In all scenarios, the current situation was applied as a comparator. PL = productivity losses due to T2D.

The results of the PSA are shown in Figure 3 in terms of population-level cost savings and QALYs gained using Scenario I as an example. The results of other scenarios are presented in Supplementary Figure S2A,B. As expected, the use of a longer time horizon increased the uncertainty related to the expected cost savings and QALY gains, leading to the wider joint distribution of cost savings and gained QALYs. However, regardless of this uncertainty, all plotted PSA iterations on an X–Y plane (Figure 3) constituted by cost savings and QALY gains remained in the southeast quadrant of the plane, where an intervention is expected to have greater effectiveness at lower costs. In addition, to take this parameter uncertainty into account, the probability of cost savings with and without T2D-related productivity loss costs conditional on the available data was estimated. Figure 4 illustrates the probabilities of cost savings in the modeled scenarios when applying the 20-year time horizon as an example. For example, as shown in Figure 4, there is around 97% probability at least for 1000 M€ savings in a case of Scenario III (when also considering the changes in productivity losses) conditional on the parameter uncertainty of the applied model. The results of other applied time horizons are presented in Supplementary Figure S3A,B. 

## 4. Discussion

The results of our study quantified the health economic significance of increased whole grain food consumption from the perspective of T2D prevention among the Finnish adult population. The inclusion of costs associated with T2D-related work absences and permanent work disabilities increased the savings potential, significantly highlighting the need for considering intervention consequences in a societal perspective in public health policy making. Our findings agree with the results of previous studies from Australia and Canada, showing a significant savings potential in the prevention of T2D among adult populations by increasing whole grain consumption [20,21,22]. However, our study demonstrated not only the significant savings potential but also significant gains in the number of QALYs (i.e., years lived in full health). This positive change in the number of years lived in full health is particularly important from the individual perspective, since the avoidance of T2D will provide life-years without T2D-related morbidity impacting negatively on an individual’s health-related quality of life [40,41,42,43,44]. In addition, a previous Finnish study has shown the relationship between the future risk for T2D and current health-related quality of life [48]. Thus, the reduced future risk for T2D could also have an immediate positive impact on an individual’s current quality of life mediated via changes in an individual’s daily dietary habits and body weight. However, for simplicity, this positive immediate effect on health-related quality of life was not considered in the present study. 

In the present study, we focused on assessing the expected population-level impacts of the hypothetical scenarios, where the proportion of Finnish adults using whole grain foods daily (i.e., at least one whole grain serving a day) is increased by a 10%-unit or alternatively where the number of daily whole grain servings is increased by one serving (i.e., two or more additional whole grain servings a day) among those who already use at least one whole grain serving a day. Based on our results, the increased whole grain consumption will lead to a higher number of health benefits and greater savings when focused on those who currently already use at least one whole grain serving a day due to the bigger size of the existing subpopulation among the Finnish adults (i.e., the majority of the Finnish adults already use at least one serving of WGF a day). However, as shown in the third scenario, the largest benefits could be expected to occur by combining these two approaches. The realization of these expected health benefits and cost savings will naturally require that public health policies supporting the increased consumption of whole grains, such as labeling, campaigns, and endorsement by manufacturers and catering services in schools, workplaces, health care, etc., can be implemented on a national level. Naturally, the implementation of such policies requires upfront investments, but these investments could be expected to be offset by the cost savings in the future with a potentially greater return on investment (ROI). However, the obtained level of ROI is conditional on an initial required investment as well as on the acceptable time horizon of that investment, since as in the case of all preventive policies, health benefits, and cost savings materialize beyond the present. Therefore, in the present study, we applied discounting to consider the fact that decisionmakers generally value future health benefits and cost savings less than current health effects and cost savings [49]. Thus, all results represent the present value of the future health and economic benefits of increased WGF consumption at the population level. Based on the conducted sensitivity analyses, the results of the study were sensitive among others to the applied annual discount rates, highlighting the need for the proper selection of discount rates to reflect societal preferences in public health policymaking. 

A particular strength of the present modeling study is that we applied nationally representative data to estimate the long-term incidence of T2D in the target subpopulations [23,28]. Furthermore, we also applied the recent results by Hu et al. [10] providing the non-linear marginal effects of an increased number of whole grain servings a day, reducing the risk for T2D. In addition, we applied Finnish estimates for the incidence of complications in patients with newly diagnosed T2D and T2D-related additional health care costs as well as nationally representative estimates for productivity losses associated with T2D and its complications. As mentioned above, the inclusion of T2D-related productivity losses had a significant impact on the obtained results. This finding agrees with recent studies highlighting the significant role of productivity losses in T2D-related economic burden [50,51]. 

As in all modeling-based studies requiring assumptions, there are also several limitations that need to be considered when interpreting the results of the present study. First, in the present study, we defined the national level of daily whole grain consumption based on a self-reported daily use of rye bread, porridge, or mixed bread observed in FinHealth 2017 [23]. We did not have information on the consumption of other whole grain products e.g., whole grain cereals or brown rice, which may have led to the underestimation of WGF consumption in the Finnish adult population at the baseline of the study. Therefore, the obtained results may be too optimistic, assuming a lower baseline population-level whole grain consumption than there really is in practice. Second, we focused on the adult population aged 30–79 years without T2D at baseline, since the risk for T2D elevates gradually after the age of 30, ignoring the long-term health and economic benefits of increased whole grain consumption in the younger Finnish population (i.e., <30 years). Third, our present study considers only a partial savings and QALY gain potential produced by increasing daily whole grain consumption, since there is well-established evidence for the benefits of whole grains, for example, in the prevention of cardiovascular diseases and various types of cancers [52,53,54]. For example, a recent study from the US showed substantial cardiovascular health care savings potential associated with increased whole grains consumption among the US adults [55]. However, since cardiovascular complications are common in the patients with T2D, the benefits obtained by reducing cardiovascular morbidity are partly considered also in the present study. Fourth, in the present study, risk factor levels for T2D were assumed to stay at the same level as they were in year 2017. This may lead to an underestimation of expected benefits due to the current unfavorable increasing trends of obesity among the Finnish adult population [56]. Finally, in the present study, we did not consider the costs of different public policies promoting the daily use of whole grain products, thus not allowing the cost-effective considerations of different policy approaches. However, we believe that the results of the present study support the development of such policies, promoting whole grain consumption and providing possibilities to assess the cost-effectiveness of such policies in the future. 

As a summary, the findings from this modeling study suggest that increased whole grain consumption could lead to significant health gains and societal savings by preventing the incidence of T2D in the Finnish adult population, even when its current daily whole grain consumption is already at relatively high level in a global context. 

## Figures and Tables

**Table 1:** Baseline characteristics of the cohorts used to define the size of the target cohort and its underlying risk of T2D in the Markov model. See Supplementary Table S1 for further details.

	Men	Women	Both
Population (not excl. T2D) (30–79 years) * [26]	1,673,290	1,702,260	3,375,550
Prevalence of T2D in whole population (HbA1c ≥ 48 or fasting glucose ≥ 7) (%) **	14.6	9.4	12.0
Estimated population size without T2D (30–79 years)	1,428,990	1,542,248	2,971,238
Estimated average age of population at baseline	53.1	54.2	53.5

* Official Statistics of Finland (OSF): Population structure [e-publication], 2018; ** Koponen et al. [23].

**Table 4:** Projected cumulative economic changes compared with the baseline situation in the year 2017 with and without productivity costs.

**Expected Savings Potential with Productivity Costs (M€) with 95% CIs; [Savings in %]**									
**Scenario ^#^**	**10-Year Time Horizon**			**20-Year Horizon**			**30-Year Horizon**		
	**Women**	**Men**	**Total**	**Women**	**Men**	**Total**	**Women**	**Men**	**Total**
Scenario I	113.0 (41.8 to 236.7)	172.5 (74.1 to 316.0)	285.5 [3.3%] (115.9 to 552.7)	341.9 (132.7 to 663.2)	486.1 (224.3 to 842.0)	828.0 [3.0%] (357.0 to 1505.2)	565.0 (279.9 to 930.7)	656.9 (345.3 to 1015.7)	1221.9 [2.6%] (625.2 to 1946.4)
Scenario II	248.0 (79.0 to 517.0)	367.6 (138.0 to 745.5)	615.6 [7.2%] (217.0 to 1262.5)	707.8 (269.2 to 1368.8)	1043.1 (430.8 to 1925.5)	1750.9 [6.6%] (699.9 to 3294.3)	1200.3 (479.9 to 2156.3)	1402.3 (661.0 to 2316.9)	2602.6 [5.7%] (1140.9 to 4473.2)
Scenario III	402.1 (153.0 to 781.5)	587.0 (235.7 to 1111.9)	989.2 [12.2%] (388.7 to 1893.4)	1145.4 (441.5 to 2281.8)	1669.6 (770.2 to 2929.4)	2815.0 [11.2%] (1211.7 to 5211.2)	1871.7 (848.7 to 3164.0)	2365.7 (1235.1 to 3694.5)	4237.3 [9.6%] (2083.8 to 6858.5)
**Expected Savings Potential without Productivity Costs (M€) with 95% CIs; [Savings in %]**									
	**10-Year Time Horizon**			**20-Year Horizon**			**30-Year Horizon**		
**Scenario ^#^**	**Women**	**Men**	**Total**	**Women**	**Men**	**Total**	**Women**	**Men**	**Total**
Scenario I	44.1 (15.2 to 91.8)	66.0 (26.2 to 125.5)	110.0 [3.4%] (41.4 to 217.2)	263.7 (102.5 to 516.7)	347.4 (174.9 to 599.3)	611.1 [3.0%] (277.4 to 1116.0)	488.5 (223.8 to 838.6)	531.2 (281.0 to 869.0)	1019.7 [2.5%] (504.8 to 1707.6)
Scenario II	91.9 (28.5 to 195.2)	136.9 (49.0 to 266.3)	228.8 [7.2%] (77.4 to 461.5)	565.7 (203.0 to 1074.8)	735.5 (310.0 to 1298.8)	1301.2 [6.4%] (512.9 to 2373.6)	1027.9 (439.0 to 1830.8)	1132.1 (533.1 to 1931.1)	2160.0 [5.4%] (972.1 to 3761.8)
Scenario III	146.1 (51.9 to 298.3)	222.1 (90.4 to 433.8)	368.2 [12.3%] (142.3 to 732.0)	909.7 (384.5 to 1665.7)	1219.2 (565.8 to 2091.2)	2128.9 [11.0%] (950.3 to 3756.9)	1678.1 (801.4 to 2871.1)	1824.0 (959.6 to 2805.2)	3502.2 [9.3%] (1760.9 to 5676.3)

^#^ Scenario I: a 10%-unit increase in the Finnish population using at least one whole grain serving a day, Scenario II: one or more additional whole grain servings a day among those who already use at least one whole grain serving a day, and Scenario III: the combination of Scenarios I and II. In all scenarios, the current situation was applied as a comparator.

**Table 5:** Projected cumulative mean QALY changes (95% CIs) compared with the baseline situation in the year 2017.

	10-Year Horizon			20-Year Horizon			30-Year Horizon		
Scenario ^#^	Women	Men	Total	Women	Men	Total	Women	Men	Total
Scenario I	501 (170 to 1041)	822 (310 to 1587)	1323 (480 to 2628)	5300 (2021 to 9990)	8314 (3224 to 15,691)	13,614 (5245 to 25,681)	20,310 (8407 to 36,205)	23,927 (9925 to 41,424)	44,237 (18,332 to 77,629)
Scenario II	1091 (331 to 2325)	1749 (570 to 3440)	2840 (901 to 5765)	11,012 (3673 to 21,294)	17,590 (6626 to 34,373)	28,602 (10,299 to 55,667)	41,850 (16,002 to 78,749)	50,842 (19,830 to 93,074)	92,692 (35,832 to 171,823)
Scenario III	1748 (593 to 3806)	2845 (1033 to 5603)	4593 (1626 to 9409)	17,620 (6882 to 34,991)	27,494 (10,632 to 52,799)	45,114 (17,514 to 87,790)	70,426 (31,723 to 124,935)	83,668 (36,171 to 148,325)	154,094 (67,894 to 273,260)

^#^ Scenario I: a 10%-unit increase in the Finnish population using at least one WGF serving a day, Scenario II: one or more additional whole grain servings a day among those who already use at least one whole grain serving a day, and Scenario III: the combination of Scenarios I and II. In all scenarios, the current situation was applied as a comparator.

## Data Availability

The data that support the findings of this study are available from the Finnish Institute for National Institute of Health and Welfare and Joint Municipal Authority for North Karelia Social and Health Services, but restrictions apply to the availability of these data, which were used under license for the current study, and so, they are not publicly available. However, data are available from the authors upon reasonable request and with the permission of the Joint Municipal Authority for North Karelia Social and Health Services and the Finnish Institute for National Institute of Health and Welfare.

## Supplementary Materials

The following are available online at https://www.mdpi.com/article/10.3390/nu13103583/s1, Figure S1: Graphical scheme of the study design, Figure S2: Results of the probabilistic sensitivity analysis using Scenario II and III. Figure S3: Probability of cumulative savings in 10 and 20-year time horizons, Table S1: The FINDRISC score distribution in the general population [23], Table S2: Coefficients of the Weibull regression for incidence of T2D, Table S3: The complications considered to be T2D-related in the Weibull regression model, Table S4: Weibull regression coefficients for the incidence of T2D-related complications, Table S5: The correlations between the Weibull regression coefficients.
