# Supplementary material for: Type 2 Diabetes-Related Health Economic Impact Associated with Increased Whole Grains Consumption among Adults in Finland
Source: Nutrients. 2021 Oct 13;13(10):3583. doi: 10.3390/nu13103583 (PMC8541656; doi:10.3390/nu13103583)
Supplement: Supplementary file 1 [file nutrients-13-03583-s001.zip › nutrients-1391925-supplementary.pdf]

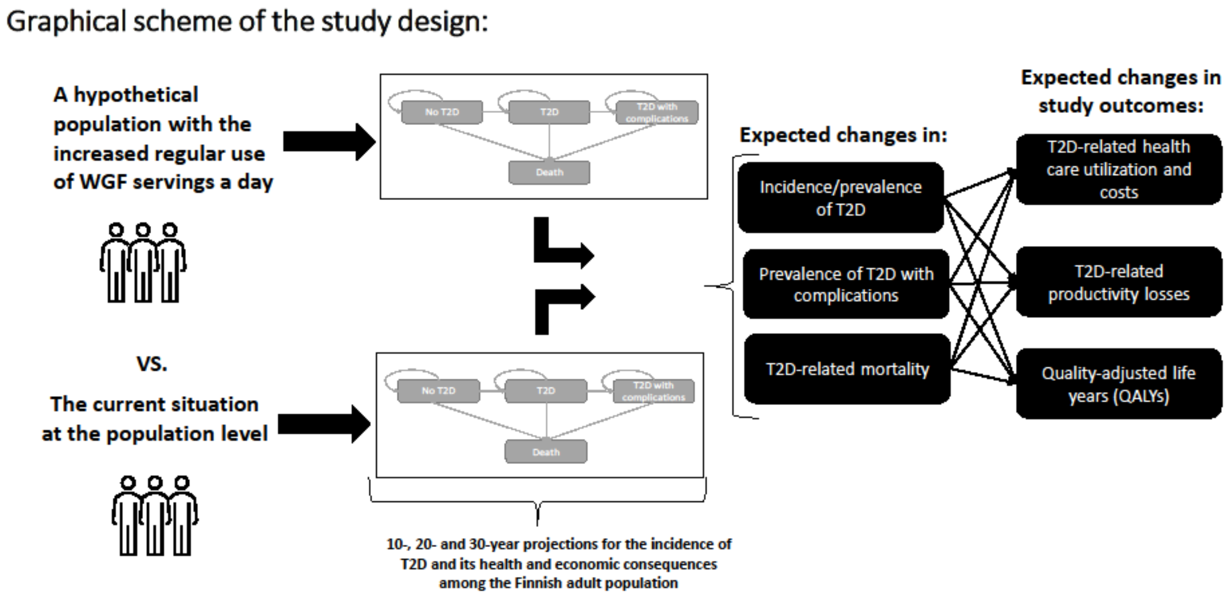

Figure S1 Graphical scheme of the study design

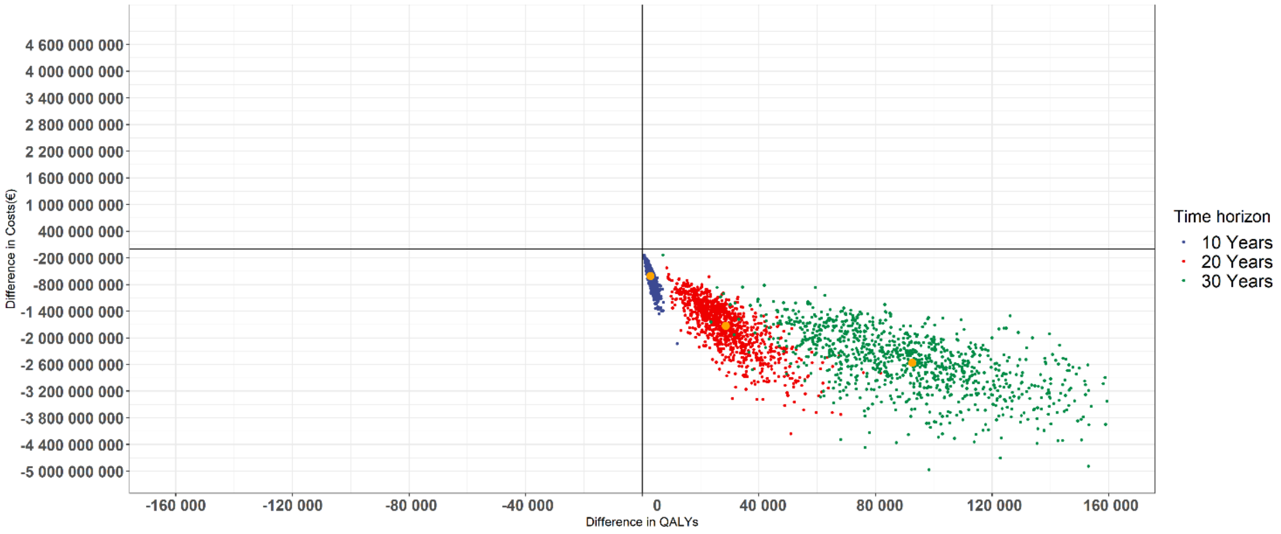

Figure S2A Results of the probabilistic sensitivity analysis showing the impact of applied time horizon on the distribution of expected population-level cost savings and gained QALYs on the X-Y-plane using Scenario II (one or more additional whole grain servings a day among those who already use at least one whole grain serving a day) as an example. Blue, red, and green color stands for 10-year, 20-year, and 30-year time horizon, respectively.

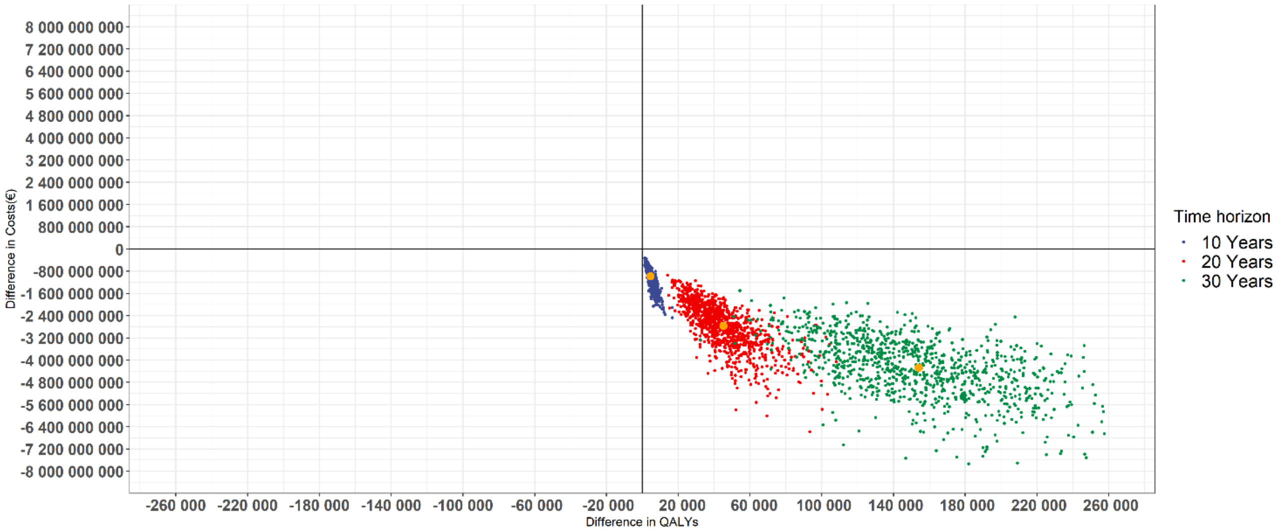

Figure S2B Results of the probabilistic sensitivity analysis showing the impact of applied time horizon on the distribution of expected population-level cost savings and gained QALYs on the X-Y-plane using Scenario III (the combination of Scenarios I and II) as an example. Blue, red, and green color stands for 10-year, 20-year, and 30-year time horizon, respectively.

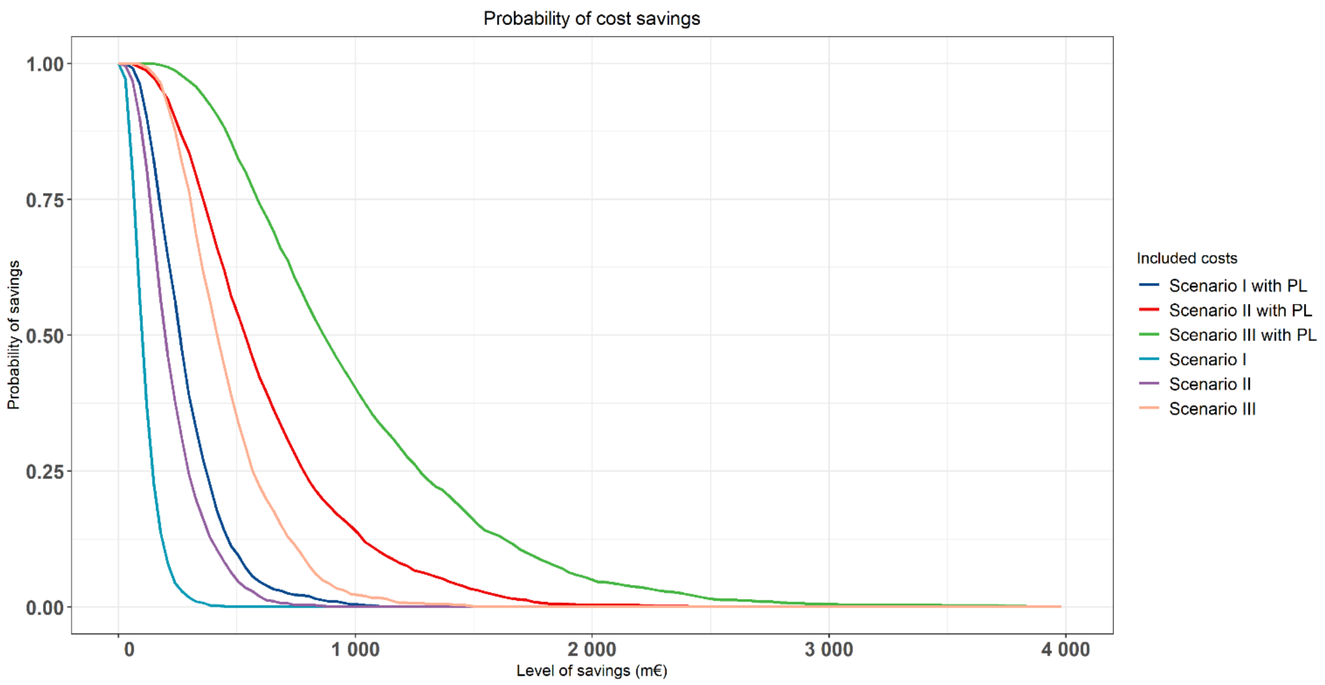

Figure S3A Probability of cumulative savings (with and without T2D-related productivity losses) in the modelled scenarios when applying the 10-year time horizon (2017 as a baseline year). Scenario I: a 10%-unit increase in the Finnish population using at least one whole grain food serving a day, Scenario II: one or more additional whole grain servings a day among those who already use at least one whole grain serving a day, and Scenario III: the combination of Scenarios I and II. In all scenarios, the current situation was applied as a comparator. PL= productivity losses due to T2D.

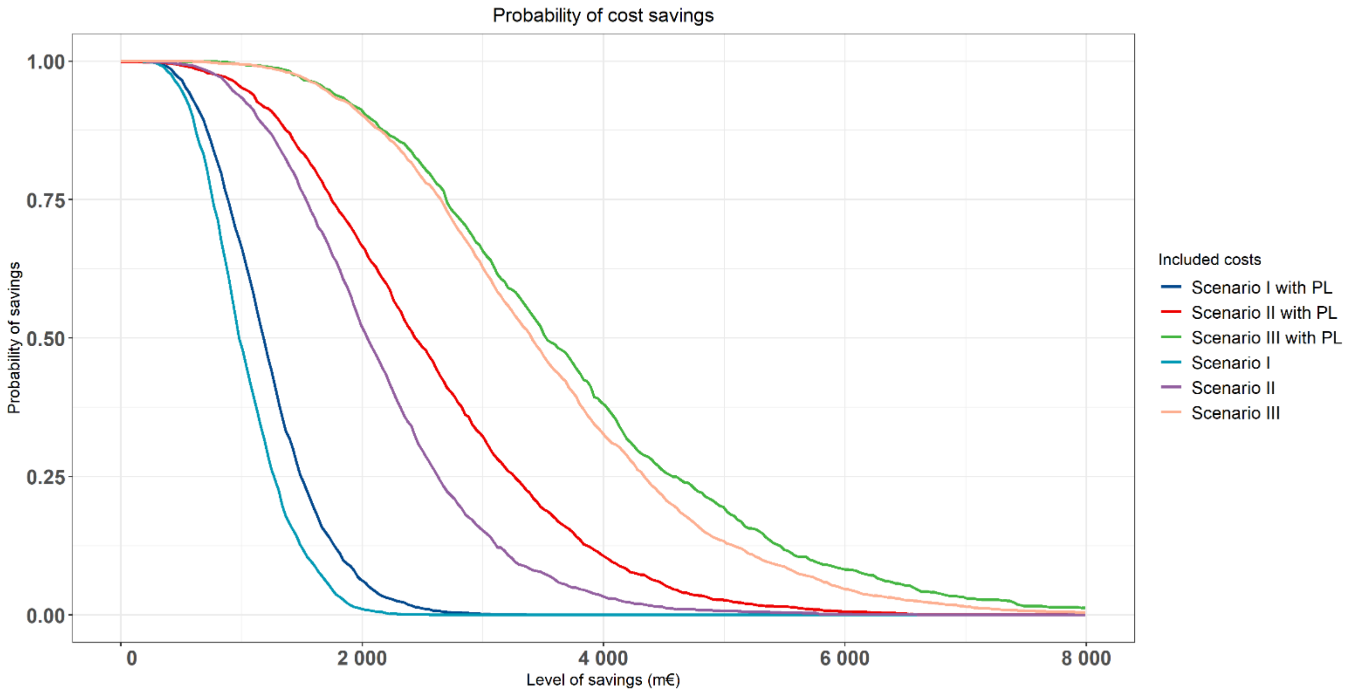

Figure S3B Probability of cumulative savings (with and without T2D-related productivity losses) in the modelled scenarios when applying the 30-year time horizon (2017 as a baseline year). Scenario I: a 10%-unit increase in the Finnish population using at least one whole grain food serving a day, Scenario II: one or more additional whole grain servings a day among those who already use at least one whole grain serving a day, and Scenario III: the combination of Scenarios I and II. In all scenarios, the current situation was applied as a comparator. PL= productivity losses due to T2D.

Table S1: The FINDRISC score distribution in the general population [23].

Gender	Age	FINDRISC 0–6	FINDRISC 7–11	FINDRISC 12–14	FINDRISC 15–19	FINDRISC 20–26
**Men**	**30 to 39**	0.738	0.214	0.048	0.00	0.00
**Men**	**40 to 49**	0.545	0.330	0.098	0.028	0.00
**Men**	**50 to 59**	0.288	0.412	0.178	0.104	0.017
**Men**	**60 to 69**	0.195	0.470	0.177	0.135	0.022
**Men**	**70 to 79**	0.193	0.484	0.155	0.143	0.025
**Women**	**30 to 39**	0.687	0.216	0.073	0.022	0.001
**Women**	**40 to 49**	0.506	0.352	0.093	0.045	0.005
**Women**	**50 to 59**	0.266	0.425	0.185	0.105	0.019
**Women**	**60 to 69**	0.163	0.444	0.237	0.126	0.003
**Women**	**70 to 79**	0.130	0.379	0.228	0.224	0.039

Table S2: Coefficients of the Weibull regression for incidence of T2D.

Parameter (95 % CI)	Value (variation)	p-value	Distribution	Distribution values used in PSA Mean (SE)
Gamma	2.329 (2.101 to 2.582)	<0.001	Normal	2.329 (0.122)
Sex Coefficient	-0.256 (-0.370 to -0.150)	<0.001	Normal	-0.256 (0.256)
Age Coefficient	-0.002 (-0.091 to 0.005)	0.532	Normal	-0.002 (0.0016)
Constant	4.668 (4.158 to 5.185)	<0.001	Normal	4.668 (1.199)
FINDRISC 0-6	0.000 (0.000 to 0.000)	Reference	Normal	0.000 (0.000)
FINDRISC 7-11	-0.486 (-0.779 to -0.192)	<0.001	Normal	-0.486 (0.691)
FINDRISC 12-14	-0.908 (-1.200 to -0.615)	<0.001	Normal	-0.908 (0.687)
FINDRISC 15-19	-1.284 (-1.569 to -0.998)	<0.001	Normal	-1.284 (0.672)
FINDRISC 20+	-1.658 (-1.964 to -1.351)	<0.001	Normal	-1.658 (0.719)

The FINDRISC score groups (0-6, 7-11, 12-14, 15-19 and 20-26) are beta coefficients of the regression model.

Table S3: The complications considered to be T2D-related in the Weibull regression model.

**Complication**	**ICD-10**
**Eye complications**	
Retinopathy or other diabetic eye complication,	E11.3, H28.0, H36 (excluding H36.8), H40.5, H42.0, H43.1, H45.0,
**Renal complications**	
Renal insufficiency	E11.2, N08.3, N18
End-stage renal disease (with or without dialysis)	Z49, Z94.0
**Neuropathic complications**	
Amputation of lower extremities	NOMESCO: NFQ10, NFQ20, NGQ10, NGQ20, NHQ10, NHQ20, NFQ48, NGQ48, NHQ30, NHQ40, NHQ60
Diabetic neuropathy, Charcot foot or other diabetic foot complication	E11.4, E11.5, E11.6, G59.0, G63.2, G73.0, G99.0, I70.2, I73.9, I79.2, L97, M14.2, M14.6, N48.4
**Cardiovascular complications**	
Angina pectoris	I20.0
Chronic heart failure	I11.0, I13.0, I13.2, I50
Incident myocardial infarction/cardiac arrest	I21, I46
Other ischemic heart disease	I22–I25
**Cerebrovascular complications**	
Ischemic stroke	I63–I66 (excluding I63.6)

Table S4: Weibull regression coefficients for the incidence of T2D-related complications.

Parameter (95 % CI)	Value (variation)	p-value	Distribution applied in PSA	Distribution values used in PSA Mean (SE)
Gamma	0.857 (0.778 to 0.944)	<0.001	Normal	0.857 (0.084)
Age coefficient	-0.062 (-0.075 to -0.049)	<0.001	Normal	-0.062 (0.063)
Sex coefficient	-0.760 (-1.355 to -0.485)	<0.001	Normal	-0.760 (0.444)
Constant	8.430 (7.290 to 9.570)	<0.001	Normal	8.430 (1.166)

PSA; Probabilistic Sensitivity Analysis

Table S5: Correlations between the Weibull regression coefficients.

	Weibull regression coefficients, risk of T2D						
	**FINDRISC 7-11**	**FINDRISC 12-14**	**FINDRISC 15-19**	**FINDRISC 20+**	**Age coefficient**	**Sex coefficient**	**Constant**
**FINDRISC 7-11**	1						
**FINDRISC 12-14**	0.838	1					
**FINDRISC 15-19**	0.837	0.885	1				
**FINDRISC 20+**	0.763	0.829	0.873	1			
**Age coefficient**	-0.017	-0.033	-0.039	-0.047	1		
**Sex coefficient**	0.042	0.058	0.125	0.213	-0.011	1	
**Constant**	-0.476	-0.527	-0.566	-0.562	-0.752	-0.21	1

	Weibull regression coefficients, risk of T2D complications		
	**Age coefficient**	**Sex coefficient**	**Constant**
**Age coefficient**	1		
**Sex coefficient**	0.284	1	
**Constant**	-0.911	-0.630	1
